# Prevalence and associated factors of recent HIV-1 infection among newly identified HIV-positive individuals tested with the Asante Rapid Recency assay in Harari region, Eastern Ethiopia

**DOI:** 10.3389/fepid.2025.1443148

**Published:** 2025-02-24

**Authors:** Muzemil Ebrahim Nure, Fitsum Weldegebreal, Fikru Tebeje, Akewok Sime, Lemma Demissie Regassa

**Affiliations:** ^1^ Moyale Primary Hospital, Oromia Health Bureau, Moyale, Ethiopia; ^2^ School Medical Laboratory Sciences, College of Health and Medical Sciences, Haramaya University, Harar, Ethiopia; ^3^ Laboratory Bacteriology Research, Department of Diagnostic Sciences, Faculty of Medicine and Health Sciences, Ghent University, Ghent, Belgium; ^4^ School of Public, College of Health and Medical Sciences, Haramaya University, Harar, Ethiopia

**Keywords:** recent HIV infection, newly identified HIV-positive, Asante Rapid Recency assay, cross-sectional study, Ethiopia

## Abstract

**Background:**

Human immunodeficiency virus (HIV) is a virus that attacks the immune system. Globally, more than 79.3 million people have been infected with it, and about 36.3 million people have died since the beginning of the epidemic. Ethiopia is one of the major affected countries in sub-Saharan Africa, with a huge number of people living with HIV. The identification of recent HIV-1 infections plays a crucial role in guiding prevention and control interventions. Moreover, data on the prevalence and factors associated with recent HIV-1 infection among cases tested by the Asante Rapid Recency Assay at health facilities in the Harari region has been inadequate. This study aimed to assess the prevalence and associated factors of recent HIV-1 infection among newly identified HIV-positive individuals tested with the Asante Rapid Recency Assay in Health Facilities of Harari Region, Eastern Ethiopia.

**Methods:**

Retrospective cross-sectional study was employed using HIV-1 diagnoses data from April 15–20, 2024 with 580 study participants. The data were extracted based on the standardized HIV Case-Based Surveillance report form, as outlined by the Ethiopian Public Health Institute. Data extracted from Redcap were checked and cleared for completeness then entered and analysed using the Statistical Package for Social Science software version 27. Bivariate and multivariable regression analyses were carried out to examine the associations between dependent and independent variables. A *P*-value of <0.05 was considered statistically significant.

**Results:**

The overall prevalence of recent HIV infection was 9.1% (95% CI: 7.0%, 11.8%). The highest proportion of recent HIV infections was in the year 2019 [9(22.5%)]. The study also found that no formal education (AOR = 18.424, 95% CI = 1.468–231.2), primary educational level (AOR = 22.1, 95% CI = 1.91–256.1, *P* = 0.013), no formal education (AOR = 18.424, 95% CI = 1.468–231.2, *P* = 0.028), having sex in the last 12 months (AOR = 5.508, 95% CI = 2.167–15.7, *P* = <.001), having sex with known/suspected HIV positive (AOR = 4.35, 95% CI = 1.455–13.04, *P* = 0.009) and Illicit drug use (AOR = 57.8, 95% CI = 16.19–207.5, *P* = <.001) had higher likelihood of having recent HIV infection.

**Conclusion:**

This study found a 9.1% proportion of recent HIV infections, indicating significant ongoing HIV transmission within the community. The study also revealed multiple risk factors for recent HIV infection, including lower educational levels, recent sexual activity, sex with high-risk partners, and drug use. This study emphasizes the significance of improving targeted HIV preventive programs.

## Introduction

Human Immunodeficiency Virus (HIV) is a worldwide public health concern that has affected millions of people. HIV is a retrovirus that spreads mostly through unsafe sexual contact, tainted blood transfusions, and sharing needles or other drug paraphernalia. HIV damages the immune system, causing gradual degeneration and the emergence of acquired immunodeficiency syndrome (AIDS). There are two main types of HIV: HIV-1 and HIV-2, with HIV-1 being the predominant variant, accounting for approximately 95% of all global HIV infections (1). Recent HIV-1 infections and their related factors are critical for accurately estimating HIV incidence, investigating HIV dissemination, and comprehending HIV transmission dynamics during a recent period (2, 3).

HIV/AIDS remains a major public health issue worldwide. In 2022, an estimated 1.3 million people were newly infected with HIV, and around 630,000 people died from AIDS-related illnesses worldwide (1). Furthermore, Sub-Saharan Africa, South and Southeast Asia, Eastern Europe, Central Asia, and Central and South America have the largest HIV/AIDS burdens, accounting for approximately 68%, 12%, 4%, and 4% of the global HIV-1-infected population, respectively. The Joint United Nations Programme on HIV/AIDS (UNAIDS) established the ″95-95-95″ targets to terminate the worldwide HIV epidemic by 2030, emphasizing the importance of early detection in these locations (4).

By the end of 2022, Ethiopia had recorded 8,257 new infections and 610,350 PLHIV (5–7). The incidence of HIV in Ethiopia varies by region, with Gambella and Addis Ababa having the highest rates. In Ethiopia, the adult HIV incidence (15–59 years old) was 0.9%, with different burdens depending on gender, age, and other demographic characteristics, as well as subregions and population groups. The urban HIV prevalence (2.9%) is about seven times greater than the prevalence in rural areas (0.4%), and women (1.2%) have twice the HIV prevalence rate as males (0.6%) (8). Efforts to control the disease are being carried out, including better testing rates, with 63% of HIV-positive youths knowing their status (9). However, challenges remain, particularly in lowering new infections and AIDS-related fatalities among young women (10). The national HIV/AIDS strategic plan for 2021–2025 aims to control the pandemic by 2025, focusing on identifying and preventing new infections. Identifying and preventing new HIV infections is crucial for achieving the global and national goals to end the HIV/AIDS epidemic by 2030 (11).

The World Health Organisation (WHO) recommends upgrading national HIV/AIDS tactical information systems by gathering longitudinal and individual-level data on newly diagnosed HIV cases. Furthermore, WHO suggests employing the Asante HIV-1 Rapid Recency Assay and HIV-1 viral load (VL) measurement as a recent infection testing algorithm (RITA). This approach aims to better understand subnational epidemics and direct specific interventions (12, 13). However, Ethiopia has hurdles due to limitations in current information systems, such as trustworthy statistics on the incidence and spread of recent HIV-1 infections. The majority of existing HIV-1 surveillance systems rely on prevalence data, which includes both past and present infections but does not capture the epidemic's dynamics. Furthermore, prevalence data are frequently aggregated at the regional or national levels, which might obscure the heterogeneity and hotspots of HIV-1 transmission within subpopulations or geographical areas. To address these drawbacks and implement WHO guidelines, Ethiopia has been incorporating the ARRA into its HIV case-based surveillance (CBS) system since 2019 (14). Moreover, data on the extent and associated variables of recent HIV-1 infection among cases tested by the ARRA at health facilities in the Harari region has been inadequate. This study aimed to assess the prevalence and associated factors related to recent HIV-1 infection among newly identified HIV-positive individuals using the Asante Rapid Recency Assay in health facilities in the Harari Region, Eastern Ethiopia.

## Methods and materials

### Study area and periods

The study was conducted in the Harari regional state health facilities using the HIV-1 case-based surveillance (CBS) system from January 1, 2019, to December 30, 2023 records were retrieved. Harari Region is one of Ethiopia's smallest regional states, approximately 510 km from Addis Ababa, Ethiopia. According to the Harari Regional Health Bureau, there are four hospitals (two public, one police, and one private), one regional laboratory and research center, eight health centers, 32 health posts, and 40 private clinics. By the end of 2023, there were seven HIV CBS sites (3 hospitals, three health centers, and one clinic), including Hiwot Fana Hospital, Jugol Hospital, Harar General Hospital, Jinela Health Centre, Arategna Health Centre, Aboker Health Centre, and FGAE Clinic (15). The data were collected from April 15 to 20, 2024.

### Study design and populations

A retrospective cross-sectional study was conducted. Newly identified HIV positives aged ≥15 and complete socio-demographic, behavioral, clinical, and ARRA test result data in HIV CBS were included in this study. The study excluded newly detected HIV positives with equivocal (not confirmed by VL test) ARRA test results as well as those with incomplete records.

### Sample size estimation and sampling technique

The sample size for the study was determined by a double population proportion formula , where $\bar{p}\;=\;p1\;+\;p2/2,\;Z_{\beta }$ was the critical value of the normal distribution at *β* (for a power of 80%, *β* was 0.2 and the critical value was 0.84) using EPI Info 7 statistical software with the assumptions of a two-sided confidence level of 95%, a power of 80%, and the ratio of exposed to unexposed 1:1, based on a recent HIV infection among newly diagnosed cases and associated factors in Amhara regional state, Northern Ethiopia (16). Therefore, the final sample size was 374. Even though the calculated sample size for the study was 374, to ensure representativeness and due to the feasibility of resources, the study included all the study subjects that fulfilled the inclusion criteria (*N* = 580) during the study period.

**Figure 1:**
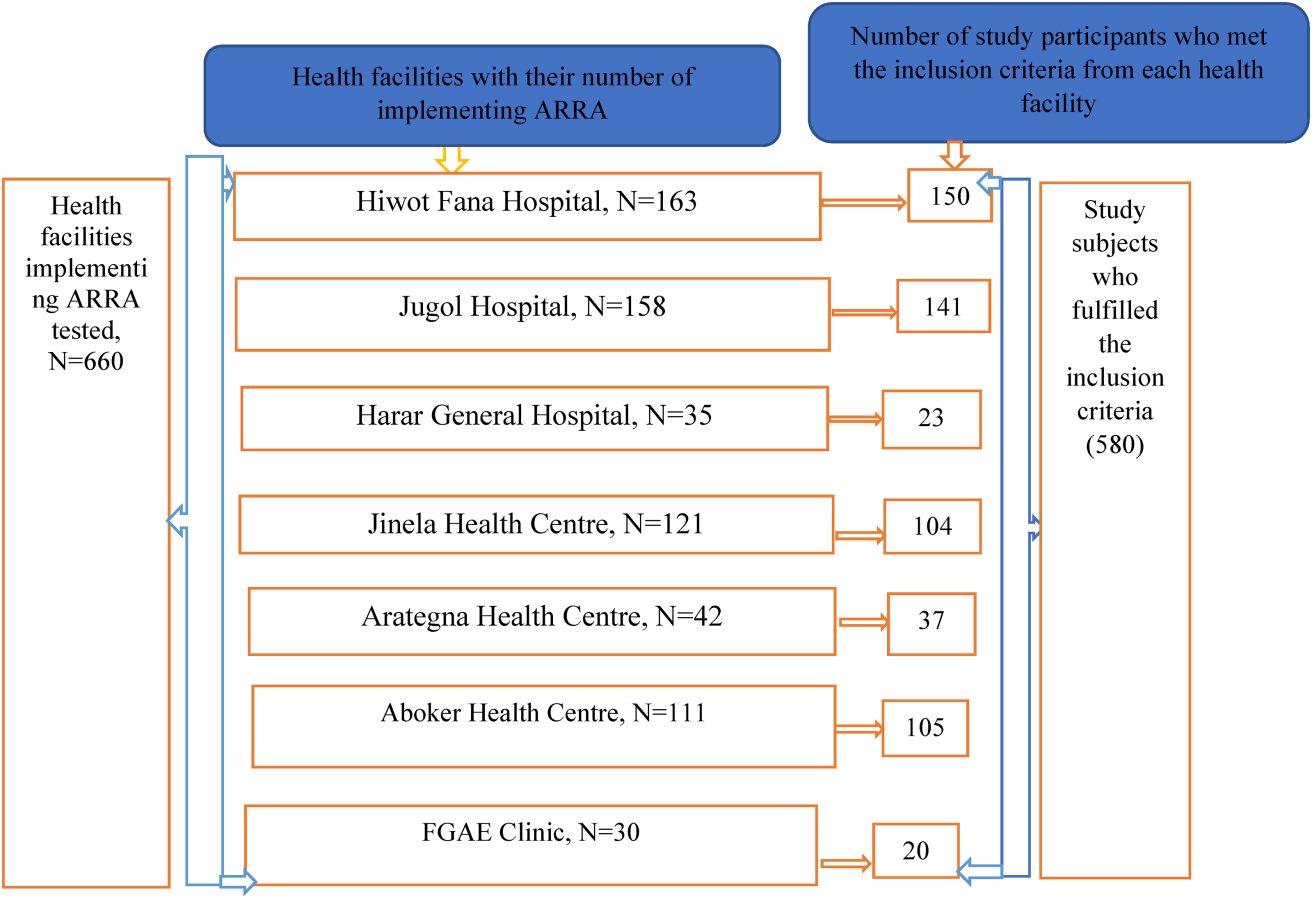
Sampling procedure for magnitude and associated factors of Recent HIV-1 Infection among newly identified HIV-positive tested with the Asante Rapid Recency assay in health facilities of Harari region, Eastern Ethiopia, 2024.

All seven health facilities in the Harari region that were implementing the HIV-1 CBS system were stratified based on patient flow, and the final sample size was allocated proportionally across health facilities during the study period. Each study participant who met the inclusion criteria was selected from each health facility using a simple random sample technique (Figure 1).

### Data collection method

Data were collected by four trained clinical nurses on HIV-1 CBS and supervised by two senior clinical nurses using a structured data extraction tool consisting of information on study subjects’ socio-demographics, baseline clinical, laboratory, risk assessment data, and ARRA test results. Various methods, including laboratory-based assays and biomarker-based examinations, were employed to diagnose recent HIV-1 infection. The Asante Rapid Recency Assay (ARRA) is one such biomarker-based examination. It is a 20-min point-of-care (POC) *in vitro* immunoassay that distinguishes recent from long-term HIV-1 infections, facilitating real-time surveillance. The ARRA identifies recent HIV-1 infections by detecting specific biomarkers indicative of early infection. It offers a fast and economical approach to identify recent cases, allowing for timely intervention and prevention efforts. The ARRA introduces fresh prospects for directing resources to geographical areas and subpopulations with an increased number of recent infections, assessing risk behaviors, improving the well-being of people living with HIV (PLHIV), and accelerating epidemic prevention (17–20).

The data extraction tool was developed based on the standardized HIV-1 CBS case report form, as outlined by the EPHI (21). The ARRA test has been evaluated and implemented in several African countries, such as Ethiopia, South Africa, Kenya, Uganda, and Tanzania, and has shown promising results in terms of performance, feasibility, and acceptability (22). Initially, data in the HIV-1 CBS system was collected at HIV testing points using a paper-based CRF, which included information on client identities, demographics, baseline clinical and laboratory data, risk assessment data, and ARRA test results for recent infection. Within 2 weeks of HIV diagnosis, health facilities enter (save) the CRF data into a web-based tool called Research Electronic Data Capture (Redcap), hosted at an EPHI server, via a secure internet connection (21). Individual-level secondary data from Redcap servers was exported into Excel format and cross-checked with a paper-based CRF from health institutions.

### Methods of data analysis

The Redcap data was extracted, validated, and cleared for completeness, and then entered and analysed with SPSS software version 27. Participants' socio-demographic and clinical features were described using descriptive statistical analysis. The association between the dependent and independent variables was determined using bivariable and multivariable logistic regression models. All explanatory variables with a *P*-value ≤ 0.25 in the bi-variable analysis were further tested via multivariable logistic regression model. Multivariable logistic regression analysis was done after adjusting for potential confounders. In the final model independent variables with *P*-values less than 0.05 at a 95% confidence interval was considered as statistically significant.

### Data quality

Data collectors were trained to ensure the accuracy, consistency, and reliability of the data they collect. Their training focuses on the specifically for data quality, including used the guidelines of ISO 8000 on how data should be collected, stored, and used to ensure integrity and quality and the HIV-1 CBS data in the Excel format was cross-checked with a paper-based case reporting form (CRF) to ensure the completeness and consistency of the data by supervisors and principal investigators. An adequate sample size was included from each study site. Moreover, data were collected following strict inclusion and exclusion criteria.

### Ethical consideration

Ethical clearance was obtained from Haramaya University's Institutional Health Research Ethics Review Committee (Ref. No. IHRERC/089/2024) and submitted to the Harari region health bureau; similarly, health bureaus wrote letters of cooperation to the selected health facilities before conducting the study. After receiving all approval letters from the responsible body, the heads of the health facility provided written and signed permission letter. The purposes of the studies were explained to the head of health facilities heads. Throughout the study period, the confidentiality of the data was strictly followed, and the collected data was not be used for another purpose that was not the intention of this study. Furthermore, this research was carried out with respect to the Helsinki Declaration.

## Results

### Socio-demographic characteristics

The study included a total of 580 newly identified HIV-positive. The age of participants ranged from 16 to 67 years, with a mean age (±SD) of 34 ± 9.8. Notably, females constituted 410 (70.7%) of the participants. Up to 91.6% of participants resided in urban area. Furthermore, 530 (91.4%), 209 (36.0%), 244 (42.1%), and 142 (24.5%) of the study participants were permanent residents, married marital, primary school in educational level, and daily laborers in occupation, respectively (Table 1).

**Table 1:** Sociodemographic characteristics of newly identified HIV-positive tested with the Asante Rapid Recency assay in health facilities of Harari region, Eastern Ethiopia, 2024 (*N* = 580).

Variables	Category	Number	Percent (%)
Age in years	15–19	19	3.3%
	20–24	63	10.9%
	25–29	126	21.7%
	30–34	138	23.8%
	35–39	89	15.3%
	40–44	55	9.5%
	45–49	38	6.6%
	≥50	52	9.0%
Gender	Female	410	70.7%
	Male	170	29.3%
Place of residence	Urban	531	91.6%
	Rural	49	8.4%
Types of residential	House/apartment/flat	557	96.0%
	Homeless	23	4.0%
Residential status	Permanent	530	91.4%
	Not permanent	50	8.6%
Marital status	Married	209	36.0%
	Divorced/separated	179	30.9%
	Never married	95	16.4%
	Widow/widower	97	16.7%
Educational level	Primary	244	42.1%
	Secondary	145	25.0%
	No formal education	127	21.9%
	Higher	64	11.0%
Occupation	Daily labourer	142	24.5%
	FSW	112	19.3%
	Unemployed/jobless	109	18.8%
	Self-business/private employed	108	18.6%
	Other^a^	24	4.1%
	Student	18	3.1%
	GO/NGO employed	67	11.6%

FSW, female sex worker.

^a^ Retried, prisoners; GO, Government Organization—a body or agency that is part of the government structure; NGO, Non-Governmental Organization—a private, non-profit organization that operates independently of the government and typically works on humanitarian or social issues. Retried, refers to an individual who has stopped working after reaching a certain age, completing their professional career. Prisoners, refers to individuals who are in prison, as a result of being convicted of a crime.

### Behavioral characteristics

Of the total of the 580 participants, 118 (20.3%) reported engaging in sexual relationships with multiple partners. In terms of sexual activity in the last 12 months, 317 (54.7%) of the participants have engaged in sexual intercourse. Regarding the participants' involvement in sex work, 125 (21.6%) of the participants indicated that selling sex had been a main source of income at some points. Additionally, 106 (18.3%) of the participants had paid or received money or gifts for sex in the last 12 months. In terms of having sex with known or suspected HIV-positive individuals, 24 (4.1%) of the participants had sex with known or suspected HIV-positive individuals in the last 12 months, and 135 (23.3%) didn't know if individuals were known or suspected HIV positive. Furthermore, 21 (3.6%) of participants had used Illicit drugs in the last 12 months (Table 2).

**Table 2:** Behavioral characteristics of newly identified HIV-positive individual tested with the Asante Rapid Recency assay in health facilities of Harari region, Eastern Ethiopia, 2024 (*N* = 580).

Variables	Category	Number	Percent (%)
Multiple sexual partners	Yes	118	20.3%
	No	462	79.7%
Having sex in the last 12 months	Yes	317	54.7%
	No	263	45.3%
Selling sex was ever a main source of income	Yes	125	21.6%
	No	455	78.4%
Paid/received money/gift for sex in the last 12 month	Yes	106	18.3%
	No	474	81.7%
Having sex with known/suspect HIV positive in the last 12 month	Yes	24	4.1%
	Don't know	135	23.3%
	No	421	72.6%
Illicit drug use in the last 12 month	Yes	21	3.6%
	No	559	96.4%

### Clinical characteristics

Among the total of 580, 217 (37.4%) of the cases were diagnosed in the OPD, followed by VCT [131 (22.6%)]. Additionally, 88 (15.2%) of the cases were diagnosed among key populations (KP) from the community in outreach outlets. Regarding prior HIV testing history; 501 (86.4%) of the participants reported were not tested for HIV before. Furthermore, a very small percentage [3 (0.5%)] of participants reported having been on pre-exposure prophylaxis (PreEP) before. Regarding disease progression, 367 (63.3%) of participants were categorized as WHO stage I. About 225 (38.8%) of the participants presented with opportunistic infections during diagnosis (Table 3).

**Table 3:** Clinical characteristics of newly identified HIV-positive individual tested with the Asante Rapid Recency assay in health facilities of Harari region, eastern Ethiopia, 2024 (*N* = 580).

Variables	Category	Number	Percent (%)
Point HIV testing the cases were diagnosed	OPD	217	37.4%
	VCT	131	22.6%
	KP from community	88	15.2%
	Triage	31	5.3%
	Other outlets^a^	113	19.5%
Receive any invasive medical procedure in the last 12 month	Yes	14	2.4%
	No	566	97.6%
Ever been tested for HIV before	Yes	79	13.6%
	No	501	86.4%
Ever been on PreEP before	Yes	3	0.5%
	No	577	99.5%
WHO stage	I	367	63.3%
	II	62	10.7%
	III	121	20.9%
	IV	30	5.2%
Opportunistic infection	Yes	225	38.8%
	No	355	61.2%

^a^ Inpatient, labour and delivery, ANC, TB clinic.

### Trends in the prevalence of recent HIV-1 infection

**Figure 2:**
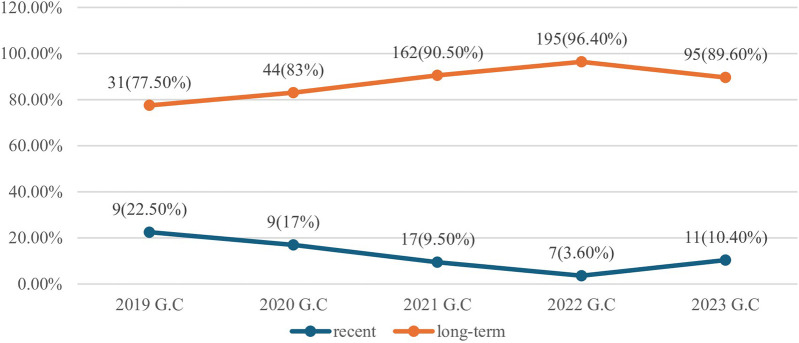
Trends of magnitude of recent HIV-1 infection among newly identified HIV-Positives tested with the Asante Rapid Recency Assay in health facilities of Harari region, Eastern Ethiopia, 2024 (*N*=580).

Among a total of 580 newly identified HIV-Positive who were tested for HIV recency test using the Asante Rapid Recency Assay in health facilities implementing HIV CBS in the Harari Region, the magnitude of Recent HIV infection was 53 (9.1%) (95%CI: 7%, 11.8%) and Long-Term HIV infection were 527 (90.9%) (Figure 2).

### Prevalence of recent HIV-1 infection

The overall prevalence of Recent HIV infection was 53 (9.1%) (95%CI: 7%, 11.8%) and long-term HIV infection were 527 (90.9%). About 14 (66.7%) of individuals reported illicit drug use, and individuals having sex with known/suspected HIV positive in the last 12 months [8 (33.3%)] had the highest proportion of recent HIV infections. The 15–19 age group [5 (26.3%)], females [38 (9.3%)], living in rural areas [5 (10.2%)], married individuals [22 (10.5%)], individuals with primary education level [32 (13.1%)] and no formal education [13 (10.2%)] had the highest proportion of recent HIV infections. Daily laborers exhibited the highest proportion of “recent” cases with 19 (13.4%), followed by female sex workers [9 (8.0%)]. Furthermore, individuals who reported having sex in the last 12 months had a substantially higher proportion of recent cases with 42 (13.2%), and individuals who reported ever relying on selling sex as a main source of income displayed a slightly higher proportion of recent HIV infection with 13 (10.4%). The highest proportion of recent HIV infections among the newly identified HIV positives tested for recency test was in the year 2019 [9 (22.5%)] followed by 2020 [9 (17%)] (Table 4).

**Table 4:** Prevalence and associated risk factors of recent HIV-1 infection Among newly identified HIV-positive individuals tested with the Asante Rapid Recency assay in Harari region, Eastern Ethiopia, 2024 (*N* = 580).

Variables	Category	HIV recency status		Total
		Long-term	Recent	
Age in years	15–19	14 (73.7%)	5 (26.3%)	19 (3.3%)
	20–24	59 (93.7%)	4 (6.3%)	63 (10.9%)
	25–29	110 (87.3%)	16 (12.7%)	126 (21.7%)
	30–34	125 (90.6%)	13 (9.4%)	138 (23.8%)
	35–39	83 (93.3%)	6 (6.7%)	89 (15.3%)
	40–44	49 (89.1%)	6 (10.9%)	55 (9.5%)
	45–49	35 (92.1%)	3 (7.9%)	38 (6.6%)
	≥50	52 (100.0%)	0 (0.0%)	52 (9.0%)
Gender	Female	372 (90.7%)	38 (9.3%)	410 (70.7%)
	Male	155 (91.2%)	15 (8.8%)	170 (29.3%)
Place of residence	Urban	483 (91.0%)	48 (9.0%)	531 (91.6%)
	Rural	44 (89.8%)	5 (10.2%)	49 (8.4%)
Types of residential	House/apartment/flat	505 (90.7%)	52 (9.3%)	557 (96.0%)
	Homeless	22 (95.7%)	1 (4.3%)	23 (4.0%)
Residential status	Permanent	485 (91.5%)	45 (8.5%)	530 (91.4%)
	Not permanent	42 (84.0%)	8 (16.0%)	50 (8.6%)
Marital status	Widow/widower	89 (91.8%)	8 (8.2%)	97 (16.7%)
	Married	187 (89.5%)	22 (10.5%)	209 (36.0%)
	Divorced/separated	165 (92.2%)	14 (7.8%)	179 (30.9%)
	Never married	86 (90.5%)	9 (9.5%)	95 (16.4%)
Educational level	No formal education	114 (89.8%)	13 (10.2%)	127 (21.9%)
	Primary	212 (86.9%)	32 (13.1%)	244 (42.1%)
	Secondary	138 (95.2%)	7 (4.8%)	145 (25.0%)
	Above secondary	63 (98.4%)	1 (1.6%)	64 (11.0%)
Occupation	Daily labourer	123 (86.6%)	19 (13.4%)	142 (24.5%)
	FSW	103 (92.0%)	9 (8.0%)	112 (19.3%)
	Self/private employee	100 (92.6%)	8 (7.4%)	108 (18.6%)
	Unemployed/jobless	98 (89.9%)	11 (10.1%)	109 (18.8%)
	Other	23 (95.8%)	1 (4.2%)	24 (4.1%)
	Student	15 (83.3%)	3 (16.7%)	18 (3.1%)
	GO/NGO	65 (97.0%)	2 (3.0%)	67 (11.6%)
Multiple sexual partners	No	419 (90.7%)	43 (9.3%)	462 (79.7%)
	Yes	108 (91.5%)	10 (8.5%)	118 (20.3%)
Having sex in the last 12 months	No	252 (95.8%)	11 (4.2%)	263 (45.3%)
	Yes	275 (86.8%)	42 (13.2%)	317 (54.7%)
Selling sex was ever a main source of income	No	415 (91.2%)	40 (8.8%)	455 (78.4%)
	Yes	112 (89.6%)	13 (10.4%)	125 (21.6%)
Paid/received money/gift for sex in the last 12 month	No	433 (91.4%)	41 (8.6%)	474 (81.7%)
	Yes	94 (88.7%)	12 (11.3%)	106 (18.3%)
having sex with known/suspect HIV positive in the last 12 month	No	387 (91.9%)	34 (8.1%)	421 (72.6%)
	Yes	16 (66.7%)	8 (33.3%)	24 (4.1%)
	Don't know	124 (91.9%)	11 (8.1%)	135 (23.3%)
Illicit drug use	No	520 (93.0%)	39 (7.0%)	559 (96.4%)
	Yes	7 (33.3%)	14 (66.7%)	21 (3.6%)
Point HIV testing the cases were diagnosed	OPD	191 (88.0%)	26 (12.0%)	217 (37.4%)
	VCT	114 (87.0%)	17 (13.0%)	131 (22.6%)
	KP from community	84 (95.5%)	4 (4.5%)	88 (15.2%)
	Triage	30 (96.8%)	1 (3.2%)	31 (5.3%)
	Other outlets	108 (95.6%)	5 (4.4%)	113 (19.5%)
Receive any invasive medical procedure in the last 12 months	No	517 (91.3%)	49 (8.7%)	566 (97.6%)
	Yes	10 (71.4%)	4 (28.6%)	14 (2.4%)
Ever been tested for HIV before	No	456 (91.0%)	45 (9.0%)	501 (86.4%)
	Yes	71 (89.9%)	8 (10.1%)	79 (13.6%)
Ever been on PreEP before	No	525 (91.0%)	52 (9.0%)	577 (99.5%)
	Yes	2 (66.7%)	1 (33.3%)	3 (0.5%)
WHO stage	I	329 (89.6%)	38 (10.4%)	367 (63.3%)
	II	59 (95.2%)	3 (4.8%)	62 (10.7%)
	III	110 (90.9%)	11 (9.1%)	121 (20.9%)
	IV	29 (96.7%)	1 (3.3%)	30 (5.2%)
Opportunistic infection	No	317 (89.3%)	38 (10.7%)	355 (61.2%)
	Yes	210 (93.3%)	15 (6.7%)	225 (38.8%)

### Associated factors

In the bivariate analysis age, residential status, educational level, occupation, having sex in the last 12 months, having sex with known/suspected HIV-positive individuals in the last 12 months, illicit drug use, ever being on pre-exposure prophylaxis (PrEP) before and received any invasive medical procedure were associated with recent HIV infection at *P* value less than 0.25 and considered for multivariable analysis. The multivariable analysis revealed educational level, occupation, having sex in the last 12 months, having sex with known/suspected HIV-positive individuals in the last 12 months, illicit drug use, and point of HIV testing remain statistically significant at *p*-value less than 0.05.

Individuals with a primary educational level (AOR = 22.1, 95% CI = 1.91–256.1, *P* = 0.013),) and individuals with no formal education (AOR = 18.424, 95% CI = 1.468–231.2, *P* = 0.028), had a significantly higher likelihood of recent HIV infection compared to those with an above-secondary educational level. Participants who reported having sex in the last 12 months (AOR = 5.508, 95% CI = 2.167–15.7, *P* = <.001), had a significantly higher likelihood of recent HIV infection compared to their sexually inactive counterparts. Participants who reported having sex with known/suspected HIV-positive individuals in the last 12 months (AOR = 4.35, 95% CI = 1.455–13.04, *P* = 0.009) had also a significantly higher likelihood of recent HIV infection compared to those who did not. Individuals who reported drug use (AOR = 57.8, 95% CI = 16.19–207.5, *P* = <.001) had a significantly higher likelihood of recent HIV infection compared to non-users (Table 5).

**Table 5:** Bivariate and multivariable binary logistic analysis of recent HIV-1 infection among newly identified HIV-positive individual tested with the Asante Rapid Recency assay in health facilities of Harari region, Eastern Ethiopia, 2024 (*N* = 580).

Variables	HIV recency status		COR (95% CI)	*P*-value	AOR (95% CI	*p*-value
	Long-term *N* (%)	Recent *N* (%)				
Age in years						
15–24	73 (89%)	9 (11%)	3.575 (0.93, 13.698)	.063*	1.214 (.222, 6.64)	0.823
25–34	235 (89%)	29 (11.0%)	3.579 (1.063, 12.05)	.040*	2.05 (.51, 8.312)	0.310
35–44	132 (91.7%)	12 (8.3%)	2.63 (0.723, 9.614)	.142*	2.36 (.554, 10.061)	0.246
>45	87 (96.7%)	3 (3.3%)	1		1	
Gender						
Female	372 (90.7%)	38 (9.3%)	1.056 (0.564, 1.975)	0.866		
Male	155 (91.2%)	15 (8.8%)	1			
Place of residence						
Urban	483 (91.0%)	48 (9.0%)	0.875 (0.331, 2.310)	0.787		
Rural	44 (89.8%)	5 (10.2%)	1			
Types residential						
House/apartment/flat	505 (90.7%)	52 (9.3%)	2.265 (0.299, 17.15)	0.428		
Homeless	22 (95.7%)	1 (4.3%)	1			
Residential status						
Not permanent	42 (84.0%)	8 (16.0%)	2.053 (0.908, 4.640)	0.084*	2.229 (0.835, 5.95)	0.11
Permanent	485 (91.5%)	45 (8.5%)	1		1	
Marital status						
Married	187 (89.5%)	22 (10.5%)	1.309 (0.561, 3.055)	0.534		
Divorced/separated	165 (92.2%)	14 (7.8%)	0.944 (0.381, 2.336)	0.901		
Never married	86 (90.5%)	9 (9.5%)	1.164 (0.429, 3.157)	0.765		
Widow/widower	89 (91.8%)	8 (8.2%)	1			
Educational level						
Primary	212 (86.9%)	32 (13.1%)	9.51 (1.274, 70.981)	.028*	22.11 (1.91, 256.01)	.013**
Secondary	138 (95.2%)	7 (4.8%)	3.196 (.385, 26.527)	.282	3.960 (.341, 46.02)	.233
No formal education	114 (89.8%)	13 (10.2%)	7.184 (.918, 56.203)	.060*	18.42 (1.468, 231.2)	.028**
Above secondary	63 (98.4%)	1 (1.6%)	1			
Occupation						
Daily labourer	123 (86.6%)	19 (13.4%)	5.02 (1.134, 22.224)	.034*	0.562 (.038, 8.39)	.676
FSW	103 (92.0%)	9 (8.0%)	2.840 (.595, 13.559)	.191*	0.983 (.161, 6.016)	.985
Self/private employee	100 (92.6%)	8 (7.4%)	3.648 (.783, 16.998)	.099*	0.167 (.021, 1.34)	.088
Unemployed/jobless	98 (89.9%)	11 (10.1%)	2.600 (.535, 12.631)	.236*	0.612 (.092, 4.091)	.612
Other	23 (95.8%)	1 (4.2%)	1.413 (.122, 16.327)	.782	0.564 (.080, 3.99)	.569
Student	15 (83.3%)	3 (16.7%)	6.500 (.997, 42.394)	.050*	8.682 (.806, 93.49)	.075
GO/NGO	65 (97.0%)	2 (3.0%)	1		1	
Multiple sex partner						
Yes	108 (91.5%)	10 (8.5%)	.902 (.439, 1.853)	.779		
No	419 (90.7%)	43 (9.3%)	1			
Having sex in the last 12 months						
Yes	275 (86.8%)	42 (13.2%)	3.499 (1.763, 6.944)	.000*	5.508 (2.167, 15.7)	<.001**
No	252 (95.8%)	11 (4.2%)	1		1	
Selling sex was ever a main source of income						
Yes	112 (89.6%)	13 (10.4%)	1.204 (.623, 2.329)	.581		
No	415 (91.2%)	40 (8.8%)	1			
Paid/received money/gifts for sex						
Yes	94 (88.7%)	12 (11.3%)	1.348 (.682, 2.663)	.390		
No	433 (91.4%)	41 (8.6%)	1			
Having sex with known/suspect HIV positive in the last 12 months						
Yes	16 (66.7%)	8 (33.3%)	5.691 (2.27, 14.256)	.000*	4.35 (1.455, 13.04)	.009**
Don't know	124 (91.9%)	11 (8.1%)	1.010 (.497, 2.052)	.979	0.903 (0.351, 2.324)	.832
No	387 (91.9%)	34 (8.1%)	1		1	
Illicit drug use						
Yes	7 (33.3%)	14 (66.7%)	26.67 (10.17, 69.92)	.000*	57.80 (16.19, 207.5)	<.001**
No	520 (93.0%)	39 (7.0%)	1		1	
Received any invasive medical procedure						
Yes	10 (71.4%)	4 (28.6%)	4.22 (1.276, 13.956)	.018*	3.70 (0.573, 23.83)	.169
No	517 (91.3%)	49 (8.7%)	1		1	
Ever been tested for HIV before						
Yes	71 (89.9%)	8 (10.1%)	1.142 (.517, 2.522)	.743		
No	456 (91.0%)	45 (9.0%)	1			
Ever been on PrEP before						
Yes	2 66.7%)	1 (33.3%)	5.048 (.450, 56.617)	.189*	0.77 (0.016, 37.54)	.894
No	525 (91.0%)	52 (9.0%)	1		1	

Key: 1: reference category; AOR, adjusted odds ratio; COR, crude odds ratio.

* Statistically significant at *p*-value < 0.25.

** Statistically significant at *p*-value < 0. 05.

## Discussion

Recent HIV infection remains a significant public health concern globally, and understanding its prevalence and associated factors is crucial for effective prevention and intervention strategies. This study aimed to investigate the prevalence of recent HIV-1 infection and identify factors associated with it among newly identified HIV-positive individuals in the Harari region, Eastern Ethiopia. Our findings contribute to the existing literature on recent HIV infections by providing evidence-based insights into the prevalence and associated factors specific to the Harari region. The study found the magnitude of recent HIV infection was 9.1% (95% CI: 7.0%, 11.8%), and associated factors such as primary educational level, no formal education, having sex in the last 12 months, having sex with known/suspected HIV positive, and Illicit drug use.

This study found the prevalence of recent HIV infection in the Harari region was 9.1%, indicating a significant ongoing transmission of HIV within the community despite the ongoing prevention efforts. The study finding also shows gaps in achieving the national HIV/AIDS strategic plan for 2021–2025 to control transmission of HIV by 2025 (23). Additionally, it also shows gaps in attaining the global objectives to halt the HIV/AIDS epidemic by 2030 (4). The 9.1% proportions in this study also align with the 8.6% finding from a study in Kenya (24), the 7% finding from a study in Lesotho (25), and the finding from Yunnan, China, which identified a proportion of 9.3% of recent infections (3). Even though the 9.1% finding in this study was lower than the findings of a previous study conducted in the Amhara region, Ethiopia, which identified a proportion of 14.2% of recent infections, it aligns in indicating ongoing transmission of HIV across different regions in Ethiopia. This underscores the importance of strengthening the targeted HIV prevention interventions to identify and address the factors contributing to recent HIV infections in the region and across different regions in the country. The slight difference in proportion can be due to the differences in population demography of the two regions.

On the other hand, the proportion finding of this study was lower than the 17% of a study conducted in Uganda (26). The 9.1% proportion finding of this study was higher than the 6.8% finding from study in Zimbabwe (27), the 6.1% finding from study in Rwanda (28), and the 3.5% finding from study in Malawi (29). This difference in the proportions of recent HIV infections might be due to variations in HIV incidence in the corresponding countries or might be due to differences in HIV recency testing algorithms used to identify recent HIV infections.

Our study identified several factors significantly associated with recent HIV-1 infection. Individuals with a primary educational level showed a higher likelihood of having a recent HIV infection compared to those with an above-secondary educational level. Additionally, Individuals with no formal education also showed a higher likelihood of having recent HIV infection compared to those with an above-secondary educational level. This finding was inconsistent with a previously conducted one in the Amhara region which found out having secondary and above education had a higher likelihood of having recent HIV infection, suggesting a potential gap in HIV prevention knowledge and risk reduction behaviours among individuals with lower educational backgrounds in the region (16). This difference could be due to variations in the study populations in terms of HIV prevention knowledge and risk reduction behaviours among individuals with different educational backgrounds. It's plausible that individuals with lower educational levels may have limited access to accurate information about HIV transmission and prevention methods, leading to higher vulnerability in the region. Furthermore, develop targeted HIV education campaigns using simple, culturally relevant language and visuals to reach individuals with lower educational attainment. And partner with education, healthcare, and social service sectors to address the intersection of low education and HIV risk comprehensively.

Furthermore, this study revealed significant associations between sexual behaviour and HIV recency status. Having sex in the last 12 months demonstrated a higher likelihood of having a recent HIV infection compared to those who were not. Additionally, engaging in sexual intercourse with known/suspected HIV-positive individuals also showed a higher likelihood of having a recent HIV infection compared to those who were not. This finding aligns with a previously conducted study in the Amhara region which found out that having engaged in sexual in the past year and having contact with an index case increases the likelihood of recent HIV infection (16). The study finding also aligns with a study conducted in Kenya (30). This similarity in findings across studies strengthens the validity of the observed association and suggests a consistent pattern across different populations and these findings underscore the role of sexual networks and partner characteristics in HIV transmission dynamics.

Moreover, illicit drug use exhibited substantially higher odds for recent HIV infections compared to non-users. This finding aligns with a study in the Amhara region (16) and with the study conducted in 14 different African countries (31). Expand access to programs that provide sterile needles and syringes to prevent the transmission of HIV and other bloodborne infections among people who inject drugs. Additionally, provide targeted education about the heightened risk of HIV associated with drug use, focusing on safer practices and the importance of seeking testing and treatment.

### Limitations of the study

As with any cross-sectional study, this one was unable to establish a cause-and-effect relationship for the components investigated. Additionally, due to the nature of secondary data, it is difficult to ascertain the reliability of recorded data. Furthermore, the study population was limited to individuals tested with HIV recency tests, potentially missing those who haven't been tested, and in this study most of the study participants were urban resident, this may potentially lead to an underestimation of the true prevalence of recent HIV infection in the general population.

## Conclusions

This study revealed a 9.1% proportion of recent HIV infections, indicating significant ongoing HIV transmission within the community. The study also revealed multiple risk factors for recent HIV infection, including lower educational levels, recent sexual activity, sex with high-risk partners, and drug use. This study emphasizes the significance of improving targeted HIV preventive programs. This study also recommends further longitudinal studies to track HIV incidence and understand transmission dynamics in the region particularly in rural areas.

## Data Availability

The original contributions presented in the study are included in the article, further inquiries can be directed to the corresponding authors.
